# Target Product Profiles for medical tests: a systematic review of current methods

**DOI:** 10.1186/s12916-020-01582-1

**Published:** 2020-05-11

**Authors:** Paola Cocco, Anam Ayaz-Shah, Michael Paul Messenger, Robert Michael West, Bethany Shinkins

**Affiliations:** 1 Test Evaluation Group, Academic Unit of Health Economics, Leeds Institute for Health Sciences, University of Leeds, Leeds, UK; 2 Academic Unit of Primary Care, Leeds Institute for Health Sciences, University of Leeds, Leeds, UK; 3 https://www.cantest.org/; 4 Centre for Personalised Health and Medicine, University of Leeds, Leeds, UK; 5 NIHR Leeds In Vitro Diagnostic (IVD) Co-operative, Leeds, UK; 6 Leeds Institute for Health Sciences, University of Leeds, Leeds, UK

**Keywords:** Medical test, Target Product Profile, TPP, Quality by design, Diagnostic, Test characteristic

## Abstract

**Background:**

A Target Product Profile (TPP) outlines the necessary characteristics of an innovative product to address an unmet clinical need. TPPs could be used to better guide manufacturers in the development of ‘fit for purpose’ tests, thus increasing the likelihood that novel tests will progress from bench to bedside. However, there is currently no guidance on how to produce a TPP specifically for medical tests.

**Methods:**

A systematic review was conducted to summarise the methods currently used to develop TPPs for medical tests, the sources used to inform these recommendations and the test characteristics for which targets are made. Database and website searches were conducted in November 2018. TPPs written in English for any medical test were included. Based on an existing framework, test characteristics were clustered into commonly recognised themes.

**Results:**

Forty-four TPPs were identified, all of which focused on diagnostic tests for infectious diseases. Three core decision-making phases for developing TPPs were identified: scoping, drafting and consensus-building. Consultations with experts and the literature mostly informed the scoping and drafting of TPPs. All TPPs provided information on unmet clinical need and desirable analytical performance, and the majority specified clinical validity characteristics. Few TPPs described specifications for clinical utility, and none included cost-effectiveness.

**Conclusions:**

We have identified a commonly used framework that could be beneficial for anyone interested in drafting a TPP for a medical test. Currently, key outcomes such as utility and cost-effectiveness are largely overlooked within TPPs though and we foresee this as an area for further improvement.

## Background

Despite significant advances and innovation in the field of disease detection, the majority of the resulting technologies fail to be translated into tests that are used in clinical practice [1, 2]*.* For instance, it is estimated that less than 1% of novel cancer biomarkers actually reach clinical practice [3]*.* One possible driver is that the development of new tests is often driven by laboratory discoveries rather than by clinical needs [4]. Test manufacturers have to fulfil extensive evidence requirements demonstrating the fitness of their test for clinical practice [5]. A poor understanding of unmet clinical needs and the clinical pathway, within which a test will sit, will often mean that the clinical and economic benefits cannot be convincingly demonstrated and hence the test may fail to be adopted into clinical practice [1]*.*

Under the ‘Quality by design’ framework, a new product is designed with the aim of meeting pre-identified quality objectives [6]. A Target Product Profile (TPP), also known as a Quality Target Product Profile (QTPP), is a strategic document which summarises the necessary characteristics of an innovative product to address an unmet clinical need [7]. TPPs exemplify the concept of ‘beginning with the goal in mind’, establishing key features and performance specifications in advance to ensure that the new product is developed to meet specific health-related goals [7, 8]. TPPs should also be seen as ‘living’ documents that can be refined and updated as additional relevant information becomes available [7, 9].

TPPs could therefore be particularly useful when designing ‘fit for purpose’ medical tests [10]; they could be used during the development and manufacturing phase to ensure that a new test meets pre-established operational and performance requirements, in line with unmet clinical need [7]*.* Generating the required evidence for a new test can take many years and significant investment [11]. TPPs therefore have great potential to be used as guiding documents for test developers to avoid late-stage development failures and reduce research waste.

Although we are aware of some examples where TPPs have been developed for medical tests, to our knowledge, there is no formal guidance as to best practice methods. In the USA, guidance for developing TPPs is available for new pharmaceutical drugs [7]. This guidance, issued by the US Food and Drug Administration (FDA), provides an overview of the purpose and attributes of TPPs, and which requirements for a new drug should be included [7]. In this context, TPPs are used as voluntary briefing documents to stimulate discussion between the manufacturer and the FDA throughout the drug development process [7]. The TPP itself outlines specific criteria that a new drug should meet [7]. However, medical tests differ from pharmaceuticals, both in terms of their characteristics and in the indirect way in which they impact on patient health [5], and therefore, this guidance is not directly transferable to the context of medical tests.

Here we report a systematic review of the methods currently used to develop TPPs for medical tests, allowing us to (1) describe a commonly adopted methodology framework, (2) outline the test characteristics for which targets are often set and (3) identify areas requiring further methodological development.

## Methods

The protocol for this review was registered on the PROSPERO database (CRD42018115133) [12].

### Search strategy

Details of the full search strategy can be found in Additional file 1. The following electronic databases were searched: MEDLINE, EMBASE, CAB Abstract Online, CINHAL, Global Health, Scopus and Web of Science. The database search was performed in November 2018 and encompassed a combination of key terms such as ‘TPP’, ‘quality by design’, ‘QTPP’ and ‘test’.

The grey literature and websites were also searched using structured methods proposed by Godin et al. [13]. A customised Google search was conducted to identify relevant websites, and then, each of these websites was hand-searched. For each website, the internal search engine was used supplemented with hand-searching to identify potentially relevant references. Duplicates across searches were removed. This search was also conducted in November 2018. For more details, please see Additional file 1: Table 1.3, Table 1.4 and 1.5.

All searches were conducted by PC and peer reviewed by an information specialist.

### Screening

TPPs written in English for any type of medical test were included (e.g. imaging, in vitro and in vivo medical tests). There were no restrictions in terms of publication date. All publication formats were included except for newsletters and PowerPoint presentations, as these did not report the methods in sufficient detail to review them.

Endnote was used to manage references. Titles and abstracts of the retrieved references were fully screened by PC based on the inclusion criteria, of which a random 10% sample was independently screened by BS. TPPs that met the inclusion criteria at this stage or those for which it was not possible to determine eligibility based on title and abstract were then screened based on the full text. For those references where full text was not available, we contacted authors. All full texts of the eligible TPPs at this stage were screened independently by PC and BS based on the inclusion criteria. The inter-reviewer agreement rate was calculated with Cohen’s *κ* statistic. For more details, please see Additional file 3: Table 3.1 and 3.2. Where any disagreements occurred, a consensus-based discussion with the other authors (MM, RW) determined whether the reference was eligible or not.

### Data extraction and analysis

A data extraction spreadsheet was developed including basic descriptive information relating to the TPP (e.g. publication format, disease of interest, targeted clinical setting, funder, time horizon). Further to this, we extracted data on the methodology used to develop the TPP, including details of the input sources (e.g. expert consultation, review of the literature), the reported decision-making process and the stakeholders involved at each stage of the TPP development. As a common decision-making framework was apparent across the TPPs, we summarised the input sources and stakeholders involved for each phase of this process. Where stakeholders and input sources for the drafting phase were not explicitly reported for, we assessed the sources included in each TPP table and the longer descriptions of each test characteristic.

Each TPP was also assessed in terms of the transparency of reporting the adopted input sources, decision-making process and which stakeholder groups were consulted.

All data extraction was conducted independently by PC and AAS, and in case of disagreement, BS, MM and RW resolved any differences.

### Test characteristic clustering

In addition to the information above, the test characteristics reported within each TPP were extracted and de-duplicated. Based on an existing evaluation framework for tests (the ACCE framework [14]), two reviewers (BS and MM) independently categorised each of the test characteristics under the following outcomes: (1) test definition, (2) analytical performance, (3) clinical validity, (4) clinical utility, (5) regulatory legitimacy and (6) economic acceptability. The category ‘test definition’ specifies the disorder of interest, target population and purpose of the test, and thus, it overlaps with the concept of ‘unmet clinical need’. Therefore, we renamed the outcome ‘test definition’ as ‘unmet clinical need’ to better represent the type of information TPPs provide.

Analytical performance describes the ability of a test to correctly detect and measure a particular analyte (e.g. precision, trueness, analytical sensitivity and specificity, limits of detection) [15, 16]. Clinical validity is defined as ‘the ability of a device to yield results that are correlated with a particular clinical condition or a physiological or pathological process or state’ [15], whilst clinical utility represents the ability of a test to affect relevant health-related outcomes for patients (e.g. improvement in quality of life, longer lifespan) [17].

Some characteristics did not fall within any of the pre-defined categories. Three additional categories were therefore identified to accommodate these additional characteristics: (7) human factors, (8) environmental impact and (9) infrastructural requirements. Human factors are concerned with the interaction between users and devices [18]. Environmental impact encompasses a change to the environment following an interaction with the product [19]. Infrastructural requirements entail ‘the stock of the basic facilities and equipment needed for realizing a product or providing a service’ [20].

## Results

### Literature search

**Fig. 1:**
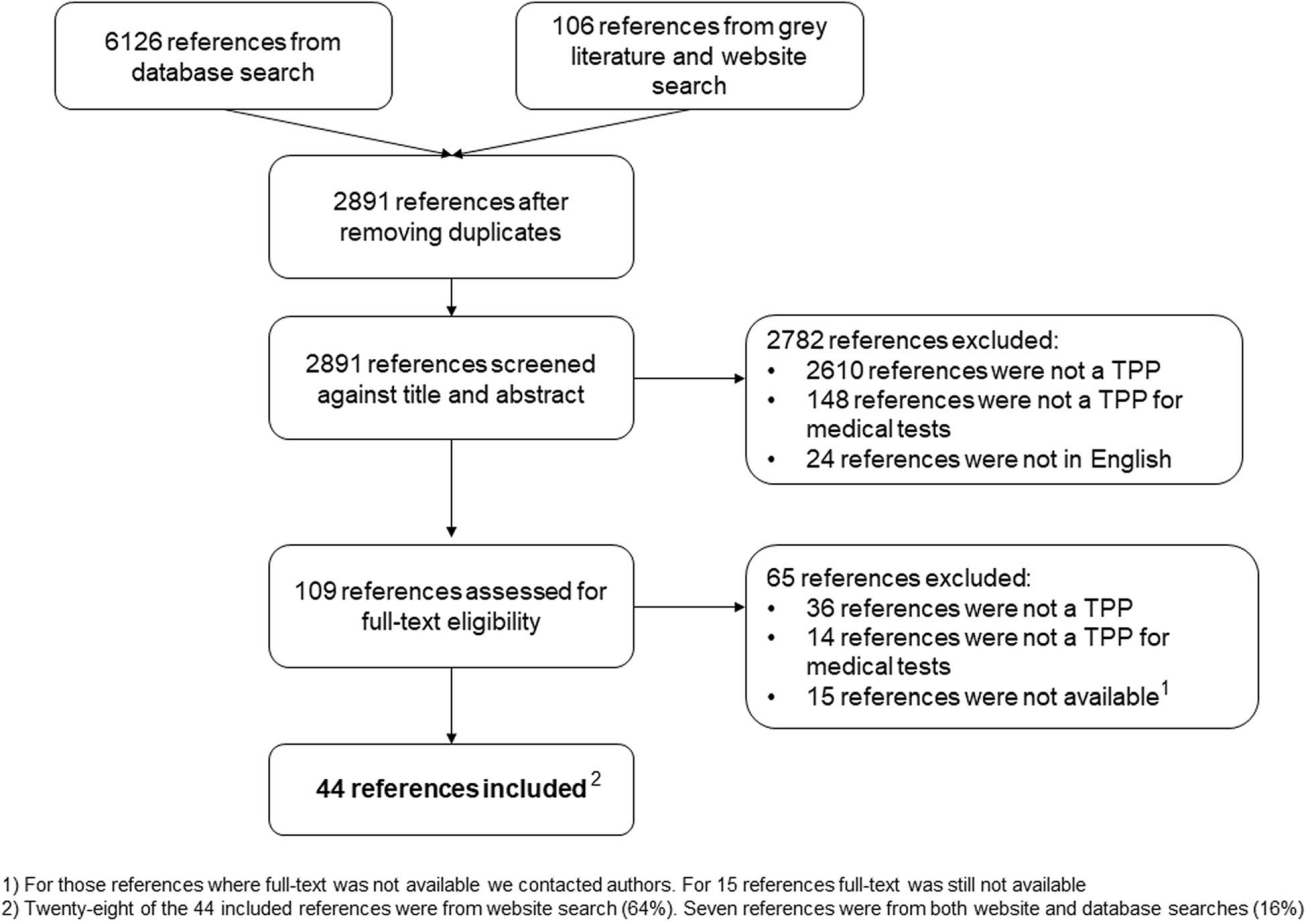
PRISMA flow diagram illustrating literature search results

Full details of the literature search results are reported in Fig. 1. Forty-four TPPs were deemed eligible for inclusion in the systematic review [8–10, 21–61]. Inter-reviewer agreement was high at title and abstract (*κ* = 96%) and full-text screening (*κ* = 98%). For more details, please see Additional file 3: Table 3.1 and 3.2.

### Features of included TPPs

The included 44 TPPs consisted of 23 reports, 16 journal articles, 4 published TPP tables (a TPP without any background information or context e.g. [34]) and one conference poster. All TPPs provided guidance on developing medical tests to detect infectious diseases. Fourteen of the 44 TPPs focused on neglected tropical diseases (32%) (e.g. soil-transmitted helminths, Chagas disease, human African trypanosomiasis, schistosomiasis, trachoma, taeniasis cysticercisosis) and on tests for vector-borne infections (32%) (e.g. Zika virus, dengue fever, hepatitis C, malaria, *E. coli*). Other types of infection included sexually transmitted infections (16%, *n* = 7), respiratory infections (14%, *n* = 6) (e.g. lower respiratory tract infection, tuberculosis, pneumonia), Ebola virus [57], meningitis [61] and severe febrile illness [8]*.*

Seven of the 44 TPPs were funded by Bill and Melinda Gates Foundation (16%), and three TPPs received funding from WHO [8, 48, 49]. The healthcare setting of interest was mostly low- and middle-income countries. The majority of TPPs did not disclose funding sources (64%, *n* = 28).

In some TPPs, a time horizon was chosen to represent the timeframe within which achieving the specifications described in the TPP was considered feasible [22, 23, 60]. In one TPP, this was based on a landscape analysis [22]. In another, expected advancements in technologies and knowledge related to a certain field seemed to justify the time horizon considered for the TPP [27]*.* Of the 44 TPPs identified, 7 reported the time horizon during which the information included in the TPP will be relevant for manufacturers (16%). Of these, 6 TPPs stated a time horizon of 5 years [22, 23, 27, 28, 51, 60], whilst the remaining considered a time horizon of 10 years [29].

### Decision-making steps

**Fig. 2:**
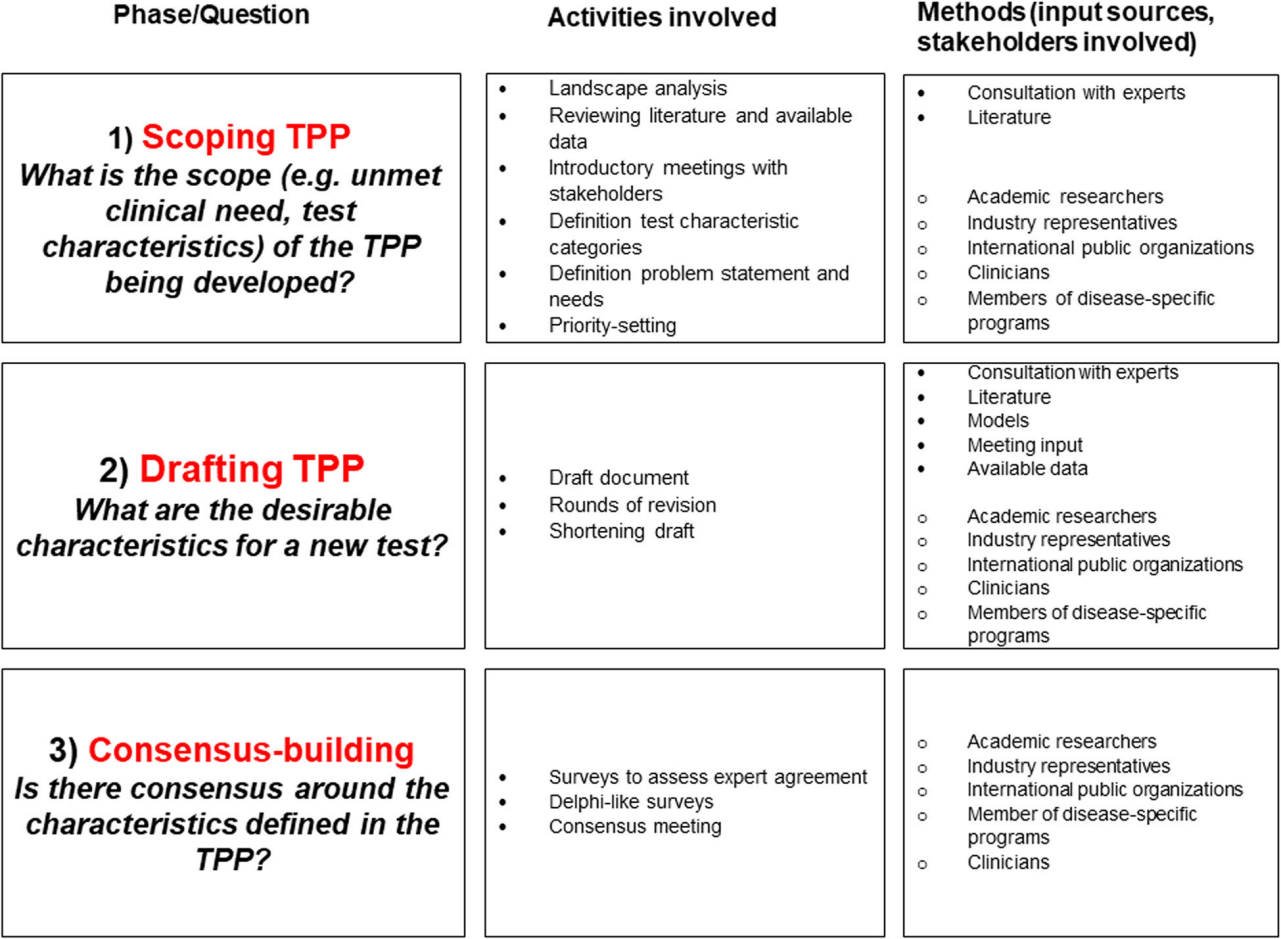
Typical activities involved, input sources and stakeholders invited for each decision-making phase

A common decision-making framework, consisting of three distinct phases, was apparent across the included TPPs: scoping, drafting and consensus-building. Figure 2 presents the most commonly adopted activities, input sources and engaged stakeholder groups.

Table 1 provides a summary of the stakeholders contributing to each phase. Some of the included TPPs are not included in Table 1 as they did not report any information related to input sources or stakeholder groups [33–36, 52, 53, 55, 61]. A summary of the input sources reported to have been used at scoping and drafting phase can be found in Additional file 3: Table 3.3.

**Table 1:** Stakeholders contributing to each phase

Stakeholder groups contributing to each phase^**a**^	Scoping	Drafting	Consensus-building
	***n*** (%)	***n*** (%)	***n*** (%)
Researchers	14 (32)	15 (34)	14 (32)
Industry representatives	11 (25)	11 (25)	14 (32)
International public organisations	9 (20)	12 (27)	12 (27)
Clinicians	7 (16)	8 (18)	8 (18)
Representatives of countries and national disease programs	5 (11)	4 (9)	8 (18)
Policy makers	4 (9)	5 (11)	4 (9)
Laboratory experts	3 (7)	5 (11)	3 (7)
Technical/funding agencies	2 (5)	3 (7)	4 (9)
Microbiologists	2 (5)	4 (9)	2 (5)
Implementers	2 (5)	2 (5)	5 (11)
Patient advocates	2 (5)	1 (2)	3 (7)
Non-profit sector	1 (2)	2 (5)	4 (9)
Modellers	1 (2)	1 (2)	1 (2)
Health economists	0 (0)	2 (5)	2 (5)
Donors	2 (5)	3 (7)	1 (2)
Market experts	2(5)	1 (2)	1 (2)
Program manager	1 (2)	2 (5)	2 (5)
Scientific associations	1 (2)	3 (7)	2 (5)
Strategists	1 (2)	1 (2)	0 (0)
Expert (unspecific)	1 (2)	3 (7)	3 (7)
Others	2 (5)	2 (5)	1 (2)

The percentages in relation to stakeholder groups do not add up to 100% because more than one stakeholder group usually contributes to the development of a TPP

^a^Percentages are calculated in relation to the total number of included TPPs (*n* = 44)

We will therefore describe the aim of each phase and breakdown the methodology (activities, input sources and stakeholders) used within the included TPPs where reported. For specific details on each included TPP, see Additional file 3: Table 3.4.

### Scoping phase methodology

Half of the TPPs provided some information on the scoping phase (*n* = 22). The aim of this phase was to provide an overview of the disease area and the limitations associated with existing technologies. The clinical problems and unmet needs were defined, in addition to identification of which test characteristics to include in the TPP.

Some of the key activities undertaken during the scoping phase included reviewing published literature (*n* = 6) or available data (*n* = 1)*,* and introductory meetings with stakeholders (*n* = 4). 

Some authors reported (*n* = 4) [22, 26, 37, 50] that they had conducted a ‘landscape analysis’, providing information on the disease area of interest, available diagnostic technologies and related characteristics and limitations. These were usually based on interviews with stakeholders and reviews of the literature. Only Toskin et al. [50] conducted a systematic literature review, reporting the databases searched and key words used.

Consultation with experts (68%, *n* = 15) and the literature (36%, *n* = 8) were the most commonly sought sources of information during the scoping phase (see Additional file 3: Table 3.3. for a full breakdown). Only one type of source was considered in 15 TPPs (of which 11 was consulting experts), whilst 7 TPPs considered more than one source.

Denkinger et al. [22] mapped the diagnostic ecosystem of interest and then performed a survey to gauge stakeholders’ preferences. Reipold et al. [48] identified the main characteristic categories (e.g. scope, performance, operational characteristics and pricing) to be included in the TPP.

Five TPPs involved a priority-setting exercise which entailed ranking each identified health need [23, 28, 32, 48, 60].

During the scoping phase, a variety of stakeholders were engaged (Table 1).

### Drafting phase methodology

The first draft of each TPP was usually prepared by either an established working group comprising experts from different organisations [9, 26, 29, 32, 40, 50, 58] or authors of the published TPP. There were two cases where the TPP was drafted by a completely different organisation [51, 57]. The TPP was often revised several times, and in some cases, it was then shortened to ensure it could be easily communicated to different stakeholders [22, 23, 28, 29, 60].

Of the 44 included TPPs, 33 of them reported which input sources were considered during the drafting phase (75%) (Additional file 3: Table 3.3). Common input sources for populating test characteristics were expert consultations (*n* = 22) and reviews of the literature (*n* = 22). Some also referred to mathematical models (*n* = 9), available data (*n* = 7), guidelines (*n* = 6) and ‘field observations’ (*n* = 5). Only one TPP was informed by pooled data from a systematic review [50].

Twenty-six of the 44 TPPs took into consideration more than one type of source at the drafting phase, as opposed to 7 TPPs which only adopted one (Additional file 3: Table 3.3). Meeting inputs were the most common single source (43%, *n* = 3).

The stakeholders engaged in the drafting phase are reported in Table 1.

### Consensus-building phase methodology

Initial agreement with the TPP was often obtained using a survey of the stakeholders (*n* = 14). The survey either included general questions regarding stakeholders views on the TPP (*n* = 4) [22, 25, 27, 51] or adopted a Delphi-like approach to provide an initial consensus on various aspects of the TPP (*n* = 10). A consensus meeting with stakeholders and experts was typically held (*n* = 11) and a revised TPP generally agreed upon. In some cases, an additional survey was sent to stakeholders on trade-offs between test attributes [48], or on rating key parameters [51, 53]. For 2 TPPs, the final TPP draft was presented to a broader stakeholder base to validate it.

The number of participants invited to the consensus-building meetings varied (< 20 participants: *n* = 5; between 20 and 50 participants: *n* = 7). One meeting included 100 participants [27]. For a few of the TPPs, the authors also took part in the consensus meetings [29, 38, 58, 60].

Less than half of the included TPPs reported information on the activities and stakeholders invited to the consensus-building phase (*n* = 19). The stakeholders engaged in the consensus-building phase are reported in Table 1.

### Transparency in reporting methods

We also assessed the transparency of the TPPs in terms of reporting their methodology (see Additional file 3: Table 3.5). The decision-making process behind the TPP was not reported in over a quarter of the included TPPs (*n* = 16). Further to this, many failed to report which information sources were considered to populate the TPP (*n* = 11). Just under half did not report which stakeholders were involved in the development of the TPP (*n* = 20). Specifically, the name of the organisations stakeholders were part was only reported in 11 TPPs, whilst 9 TPPs mentioned personal details of each stakeholder (20%) and 4 TPPs explained why certain stakeholders were invited [26, 38, 46, 58]. Sixteen TPPs reported the source of funding (36%).

There were some TPPs where the methodology was very clearly reported [26, 28, 38, 60].

### Test characteristics included in TPPs

**Fig. 3:**
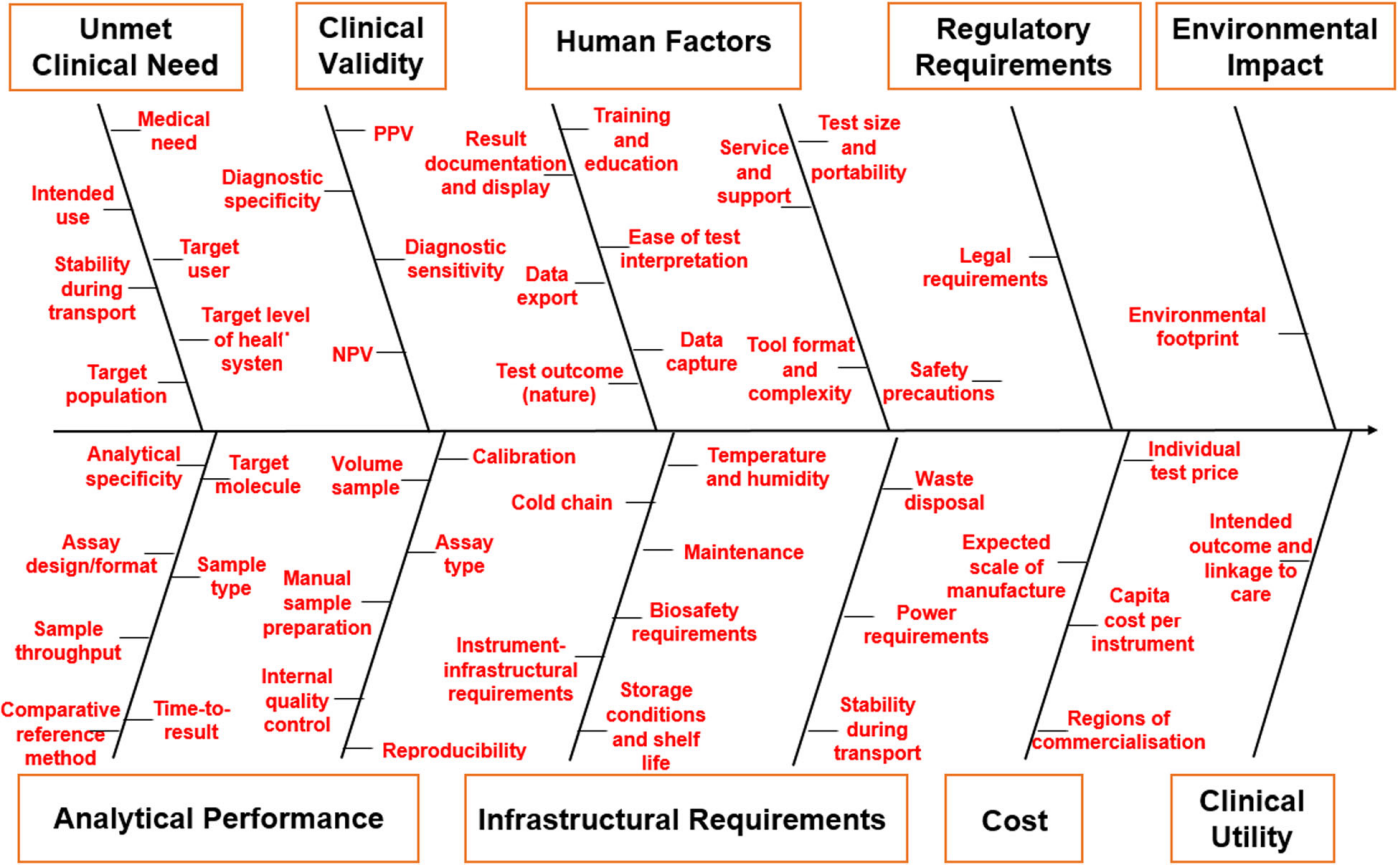
Test characteristics frequently reported in all TPPs (*n* = 44) sorted by categories

After removing duplicates, 140 different test characteristics were reported across the included TPPs. Some features which did not represent test characteristics have been excluded, such as factors relating specifically to the disease in question rather than the test. For more information, please see Test Characteristics Overview Excel spreadsheet (Availability of data and materials). Figure 3 shows the test characteristics most frequently reported (a full list is available in Additional file 2: Table 2.1).

**Fig. 4:**
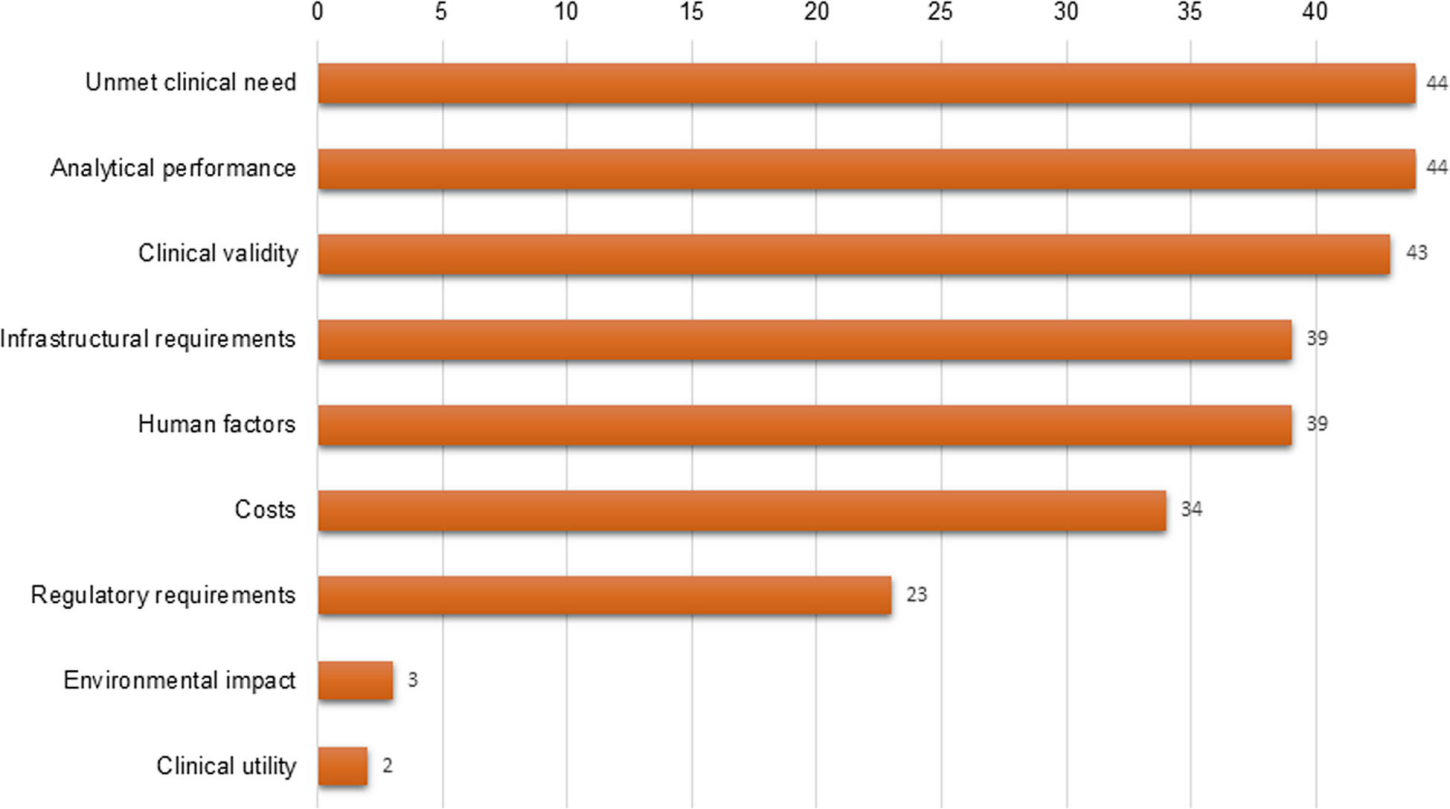
Test characteristic categories in absolute number (*n*) of included TPPs

Figure 4 depicts which characteristic categories were reported in the included TPPs. Details on unmet clinical need, analytical performance and clinical validity appeared to be consistently reported; however, regulatory requirements, environmental footprint and clinical utility were less frequently considered.

## Discussion

We report a systematic review of the methods currently used to develop Target Product Profiles for medical tests. Despite TPPs for any medical test being searched, all of the identified TPPs were focused on diagnostic tests for infection.

There was generally a lack of transparency and consistency in reporting the methods underlying TPPs. This would make it difficult to appraise the recommendations within the TPPs, ascertain whether the recommendations are generalizable to other settings, and challenging to reproduce.

### Relevancy of TPPs for test manufacturers

The purpose of a TPP is to identify, upfront, the essential characteristics of a test for it to fulfil a pre-specified, unmet clinical need. This should, in turn, increase the likelihood that the test will be adopted into clinical practice and reimbursed [1]. A TPP should also account for contextual aspects that might affect the test’s real-world performance [10], defining infrastructural and technical constraints that impact on the implementation into clinical practice. This review shows that TPPs to date have primarily been developed for global health applications, as the main funding organisations are WHO, UNICEF and Bill and Melinda Gates Foundation. The primary focus on infectious diseases may be explained by the remit of the global organisations who fund TPPs. WHO included HIV/AIDS, neglected tropical diseases, tuberculosis and malaria as priority diseases [62]. Further to this, WHO established the ‘R&D Blueprint’, which aims to promote R&D activities (tests, vaccines, medicines) during epidemics [63]. After having identified pathogen to target first, TPPs are usually commissioned to guide the development process of new healthcare products which will address the high-priority pathogen [63]*.*

However, the development of TPPs should not be limited to one specific disease area or clinical setting; the concept of ‘beginning with an end in mind’ embodied by TPPs could support both international and national health decision-makers. This activity should, in turn, stimulate innovation of new tests driven by clinical needs rather than solely by laboratory discoveries. It would also provide manufacturers with greater clarity around test requirements and confidence in the market for developing innovative tests.

### Identified limitations in current TPP methodology

In reviewing current methodology for developing TPPs for medical tests, we have identified three key areas where current TPP methodology could be improved: (1) oversight of clinical utility, (2) a focus on price rather than cost-effectiveness and (3) subjectivity of information sources. Here we discuss each limitation and the implications.

#### Oversight of clinical utility

Very few of the TPPs reported desirable characteristics relating to the clinical utility of the test. This is not surprising given that the majority of research efforts has focused on generating evidence on the analytical performance and diagnostic accuracy of a new test [11]. A highly accurate test does not necessarily mean that the test will improve patient health, as factors relating to decision-making and the effectiveness of patient management strategies could fall short [64].

Assessing the clinical utility of a new test is extremely challenging. Measuring the impact of a test on patient health outcomes is difficult as tests tend to guide patient management decisions, rather than directly impacting on patient health outcomes [5]. Therefore, estimating the clinical utility of a test requires evidence of how the information from a test is incorporated into decision-making and the downstream effectiveness of those decisions [65]. In the case of a new test, this is particularly complicated given the uncertainty around the mechanisms by which the test will impact on patient outcomes [65]*.*

#### Focus on price rather than cost-effectiveness

Although the minimum and optimal price of the tests featured in many of the TPPs, none of these was driven by the trade-off between the overall cost implications of implementing the test and the associated patient benefits. Cost-effectiveness analysis provides a framework to compare costs and benefits of an intervention against relevant comparators, including current practice. Specifically, cost-effectiveness analysis defines whether the intervention being evaluated represents good value for money.

It is important to consider the cost of the new test in the context of the benefits that the test may provide. For example, a new test may be relatively expensive but may also improve patient health to the extent that the additional is justified. Conversely, a new test may be relatively cheap but offer no improvements in patient health and therefore even the marginal increase in cost is not justified.

Conducting cost-effectiveness analysis at early R&D stages of new tests can therefore help manufacturers to avoid significant investments in tests that do not have the potential to be cost-effective [65].

These first two limitations are particularly relevant since decision-makers increasingly demand evidence that a new test improves patient health and is cost-effective rather than solely evidence of its analytical and clinical validity [5]. Specifically, many Health Technology Assessment bodies in Europe, Australia and North America consider clinical utility, cost and cost-effectiveness in relation to the target population, in addition to analytical performance and clinical validity when assessing new molecular diagnostic tests [66].

#### Subjectivity of input sources

Expert judgement and evidence identified in published literature were the main sources of information for defining desirable characteristics. Systematic reviews of the literature, where database searches are reproducible and the quality of relevant studies are appraised, were not conducted to identify relevant evidence at the scoping and drafting phase. This is likely to introduce bias and subjectivity in terms of the evidence used to underpin test characteristic recommendations.

Although expert judgement is undoubtedly useful, relying solely upon this information source has some limitations, particularly for quantitative estimates. How humans make probability judgements is highly affected by many heuristics and systematic biases (e.g. anchoring, availability, overconfidence and insight bias) [67]. Specifically, previous literature has found a poor understanding of test accuracy among healthcare professionals [68] as sensitivity and specificity are often misinterpreted and mistaken for predictive values [68].

Additionally, the quality of expert elicitation heavily relies on expert selection as it is important to choose experts with good subject knowledge. Only 4 TPPs described how the selection process took place, and therefore, the quality of expert judgements might be questioned. Furthermore, many TPPs reported literature as a source for informing TPPs; however, less than half of the TPPs cited the references considered. This lack of transparency might hinder the quality and credibility of sources on which TPPs are based.

### Study limitations

Since this study is a systematic review of publicly available literature, a key study limitation is that we have inevitably missed any confidential or unpublished TPPs developed in-house by test manufacturers. Although the results of our online searches did not identify any companies stating that they have developed TPPs for medical tests, we would not expect to find such information on company websites. Anecdotally, however, we have not encountered any formal TPP development activity (by this we mean definition of desirable test characteristics) within the National Institute for Health Research Leeds In Vitro Diagnostics Co-operative (NIHR Leeds) MIC industry network.

As there are no guidelines on how TPPs for medical tests should be developed, we did not formally assess risk of bias. We did however appraise the transparency with which the methodology underpinning each TPP had been reported. Unfortunately, it was not possible to fully evaluate the TPP developed by PATH [40] as the online appendices were not accessible. Additionally, due to poor methodological transparency, it was difficult to assess with certainty authorship of TPPs and whether authors of TPPs took part themselves in the consensus-building meetings.

### Future research

Although there is evidence of a common development framework, this review highlights that there is considerable variability in the methods employed to draft TPPs and inconsistencies in which test characteristics are described. A key issue in reviewing the methods implemented was the lack of transparency in methodology reporting.

Guidance on best practice methods for developing TPPs for medical tests would be highly beneficial. Similarly to the US FDA guidance on TPPs for drugs, a guidance document could be developed for TPPs for medical tests summarising the purpose, attributes of TPPs and which test characteristics should be included.

However, to inform the development of such guidance, future research should focus firstly on how to systematically identify unmet clinical needs underpinning a certain disease area. Monaghan et al. [69] developed a valuable checklist for identifying biomarkers based on literature findings and consultations with experts. We believe that this checklist could be pertinent for the scoping phase underlying TPP development; however, this would need further validation in this specific context.

More research is also required to understand how to better incorporate the assessment of desirable clinical utility and cost-effectiveness of innovative tests into TPPs. One possible way forward could be exploring how and if care pathway analysis and early economic modelling could be integrated into the development of TPPs. Care pathway analysis would provide clarity on the mechanisms by which a test could impact on downstream patient outcomes. Early economic modelling could be used to define desirable values for certain test characteristics (e.g. test price, diagnostic sensitivity and specificity) based on cost-effectiveness [67]. Therefore, integrating care pathway analysis and early economic modelling into TPP development might provide more evidence-based information to the test developers.

Outside of the actual methodology for developing TPPs, it would be useful to better understand whether manufacturers develop tests strictly in line with TPPs, or whether there are any factors which make this infeasible or challenging. Additionally, we would be interested in which methods manufacturers usually adopt to develop TPPs to assess if there are any differences with the methodological framework we highlighted here. To this end, interviewing test manufacturers might provide interesting insights on the intrinsic value of TPPs for the industry.

Most importantly to ensure that the development of TPPs becomes widespread practice, it would be valuable to explore how TPPs could be integrated into existing regulatory paths for innovation such as the European Union Regulation for In Vitro Diagnostics (Regulation 2017/746) and the US FDA Drug Development and Approval Process. It might then be possible to align test characteristics featured in TPPs with evidence requirements which are relevant for market approval decisions of new medical tests. This, in return, might increase the applicability of TPPs for the industry.

## Conclusions

Based on this review, we summarised current methodological practice into a framework of value to those interested in developing TPPs for medical tests.

We also identified some key weaknesses, including the quality of the information sources underpinning TPPs and failure to consider test characteristics relating to clinical utility and cost-effectiveness.

This review thus provides some recommendations for further methodological research on the development of TPPs for medical test. This work will also help to inform the development of a formal guideline on how to draft TPPs for medical tests.

## Supplementary information

**Additional file 1.** Search strategies.

**Additional file 2.** Test characteristics.

**Additional file 3.** Results.

## Data Availability

The datasets generated and analysed during the current study are available in the University of Leeds repository 10.5518/781. These datasets entail an Excel spreadsheet with data extraction and an overview of test characteristics included in reviewed TPPs.

## Abbreviations

ACCE: Analytic validity, Clinical validity, Clinical Utility, Ethical, legal and social implications
AIDS: Acquired immune deficiency syndrome
CEA: Cost-effectiveness analysis
FDA: Food and Drug Administration
FIND: The Foundation For Innovative New Diagnostics
HIV: Human immunodeficiency virus
IDC: International Diagnostics Center
NIHR: National Institute for Health Research
PRISMA: Preferred Reporting Items for Systematic Reviews and Meta-Analysis
QTPP: Quality Target Product Profile
R&D: Research and Development
TPP: Target Product Profile
UNICEF: United Nations International Children’s Emergency Fund
WHO: World Health Organization
