# Supplementary material for: Target Product Profiles for medical tests: a systematic review of current methods
Source: BMC Med. 2020 May 11;18:119. doi: 10.1186/s12916-020-01582-1 (PMC7212678; doi:10.1186/s12916-020-01582-1)
Supplement: Supplementary file 1 — Additional file 1. Search strategies. [file 12916_2020_1582_MOESM1_ESM.docx]

# **Additional File 1: Search strategies**

**Search strategy**

**Database search**

Table 1.1 Search strategy for database search

| # | Keywords |
| --- | --- |
| 10 | #8 OR #9 |
| 9 | (TPP adj5 (test* OR assay OR screen* OR exam* OR diagnos* OR analys#s)).ti,ab,kw |
| 8 | #4 AND #7 |
| 7 | #5 OR #6 |
| 6 | (test* OR assay OR screen* OR exam* OR diagnos* OR analys#s).ti,ab,kw |
| 5 | exp "Diagnostic Techniques and Procedures"/ |
| 4 | #1 OR #2 OR #3 |
| 3 | “quality by design”.ti,ab,kw |
| 2 | QTPP.ti,ab,kw |
| 1 | “target product profil*”.ti,ab,kw |

Table 1.2 List of database searched and number of references retrieved

| **Database searched** | **Date of search** | **Total number of results found (n)** |
| --- | --- | --- |
| Ovid MEDLINE ® (1946 to October Week 5 2018) | 12/11/18 | 784 |
| Ovid MEDLINE(R) In-Process & Other Non-Indexed Citations November 09, 2018 | 12/11/18 | 108 |
| Ovid MEDLINE(R) and Epub Ahead of Print (November 09 2018) | 12/11/18 | 24 |
| Embase Classic + Embase 1947 | 12/11/18 | 1896 |
| CAB Abstract online 1910 to 2018 Week 44 | 12/11/18 | 125 |
| Global Health (1910 to 2018 Week 44) | 12/11/18 | 102 |
| CINAHL | 12/11/18 | 237 |
| Scopus | 12/11/18 | 1396 |
| Web of Science Core Collection | 12/11/18 | 1454 |

**Website search**

We adopted structured methods proposed by Godin et al. (2015) in ‘Applying systematic review search methods to the grey literature: a case study examining guidelines from school-based breakfast programs in Canada’.

**Search for relevant websites**

Table 1.3 Search for relevant website results

| **Number of research** | **Date** | **Serch Engine** | **Keywords** | **# new potentially relevant records** | **Total # records** | **Notes** |
| --- | --- | --- | --- | --- | --- | --- |
| 1 | 18/10/2018 | Google | "target product profile" AND diagnostic test | 16 | 16 | Only 350 results found |
| 2 | 18/10/2018 | Google | "target product profile" AND screening | 3 | 19 | Only 308 results found |
| 3 | 18/10/2018 | Google | "target product profile" AND exam OR test OR assay | 4 | 24 | Only 304 results found |
| 4 | 18/10/2018 | Google | TPP AND diagnostic test | 0 | 24 | Only 245 results found |
| 5 | 18/10/2018 | Google | TPP AND screening | 0 | 24 | Only 332 results found |
| 6 | 18/10/2018 | Google | TPP AND exam OR test OR assay | 0 | 24 | Only 364 results found |
| 7 | 18/10/2018 | Google | QTPP AND diagnostic test | 2 | 26 | Only 119 results found |
| 8 | 18/10/2018 | Google | QTPP AND screening | 0 | 26 | Only 307 results found |
| 9 | 18/10/2018 | Google | QTPP AND exam OR test OR assay | 0 | 26 | Only 290 results found |

**List relevant websites**

Table 1.4 Detailed results of search for relevant organisations and related websites

| **# search** | **Name organization** | **URL** |
| --- | --- | --- |
| 1 | FIND | [https://www.finddx.org](https://www.finddx.org/) |
|  | WHO | [http://www.who.int](http://www.who.int/) |
|  | The International Diagnostics centre London School of Hygiene & Tropical Medicine | [http://www.idc-dx.org](http://www.idc-dx.org/) |
|  | UNICEF | [https://www.unicef.org](https://www.unicef.org/) |
|  | PATH | [https://path.org](https://path.org/) |
|  | DNDi ( Drugs for Neglected Diseases initiative) | <https://www.dndi.org/> |
|  | FDA | [https://www.fda.gov](https://www.fda.gov/) |
|  | Pink Ribbon | <http://pinkribbonredribbon.org/> |
|  | Unitaid | [https://unitaid.org](https://unitaid.org/) |
|  | Malaria consortium | <https://www.malariaconsortium.org/> |
|  | ReAct group | <https://www.reactgroup.org/> |
|  | DiaDev (Investigating diagnostics in global health) | <http://www.diadev.eu/> |
|  | Public Health Emergency | <https://www.phe.gov/> |
|  | Galvmed | [https://www.galvmed.org](https://www.galvmed.org/) |
|  | STP TB Partnership | [http://www.stoptb.org](http://www.stoptb.org/) |
| 2 | EMA | <https://www.ema.europa.eu/> |
|  | NICE | <https://www.nice.org.uk/> |
|  | IVCC | <http://www.ivcc.com/> |
| 3 | Critical Path Institute | <https://c-path.org/> |
| 4 | European Directorate for the Quality of Medicines & HealthCare | <https://www.edqm.eu/> |
| 5 | AIGHD | <https://www.aighd.org/> |
| 6 | IMPT for Reproductive Health | <https://www.theimpt.org/> |
| 7 | ICH harmonisation for better health | [https://www.ich.org](https://www.ich.org/) |
|  | Product Quality Research Institute | <http://pqri.org/> |
| 8 | N/A | N/A |
| 9 | N/A | N/A |

**Results website search**

**Keywords:** “target product profile” in Title

**Included**: documents and publications relevant for the research question based on the title

**Excluded**: PowerPoint presentation, newsletters and results not relevant for the research question based on the title

**Date search**: 18-22/10/18

Table 1.5 Details on website search and number of relevant and included references

| **Date** | **Website name** | **URL** | **Specific section searched** | **Search terms used** | **Total number of hits** | **Approach to screening for relevancy** | **Number of relevant references** | **Notes related to screening** | **Number of included references** | **Notes related to inclusion** |
| --- | --- | --- | --- | --- | --- | --- | --- | --- | --- | --- |
| 18/10/2018 | FIND | [https://www.finddx.org](https://www.finddx.org/) | Internal search engine | "target product profile" | 76 | Title | 19 |  | **22** | The number of included references entails relevant hits that were retrieved from both searches within the same website. Duplicates across both searches were deleted. |
| 18/10/2018 |  | <https://www.finddx.org/target-product-profiles/> | Hand-searched in the section of the FIND website dedicated to target product profiles | "target product profile" |  | Title | 14 |  |  |  |
| 18/10/2018 | WHO | <http://www.who.int/> | Internal search engine | "target product profile" | 19 | Title | 11 | Search for only for publications | **28** | The number of included references entails relevant hits that were retrieved from both searches within the same website. Duplicates across both searches were deleted. |
| 18/10/2018 |  | <http://apps.who.int/iris> | Internal database within the WHO website | "target product profile" | 29 | Title | 17 | Search for articles "meeting Abstracts" |  |  |
| 18/10/2018 | PATH | [https://www.path.org](https://www.path.org/) | Internal search engine | "target product profile" | 228 | Title | 24 |  | **24** |  |
| 18/10/2018 | The International Diagnostics centre London School of Hygiene & Tropical Medicine | <http://www.idc-dx.org/> | Internal search engine | "target product profile" | 8 | Title | 8 | Search for -publication -guidelines -report -white paper | **8** |  |
| 18/10/2018 |  | <http://www.idc-dx.org/themes/development-and-evaluation/target-product-profiles> | Hand-searched in the section of the website dedicated to target product profiles | "target product profile" |  | Title | 6 |  |  |  |
| 18/10/2018 | UNICEF | [https://www.unicef.org](https://www.unicef.org/) | Internal search engine | "target product profile" | 67 | Title | 7 | Search for document | **7** | The number of included references entails relevant hits that were retrieved from both searches within the same website. Duplicates across both searches were deleted. |
| 18/10/2018 |  | <https://www.unicef.org/publications/index_search.php> | Search into publication section | "target product profile" | 0 |  | 7 |  |  |  |
| 18/10/2018 | DNDi | [https://www.dndi.org](https://www.dndi.org/) | Internal search engine | "target product profile" | 27 | Title | 6 |  | **6** | The number of included references entails relevant hits that were retrieved from both searches within the same website. Duplicates across both searches were deleted. |
| 18/10/2018 |  | <https://www.dndi.org/diseases-projects/target-product-profiles/> | Hand-searched in the section of the website dedicated to target product profiles | "target product profile" |  | Title | 4 |  |  |  |
| 18/10/2018 | FDA | [https://www.fda.gov](https://www.fda.gov/) | Internal search engine | "target product profile" | 69 | Title |  |  | **0** |  |
| 18/10/2018 | Pink ribbon | [http://pinkribbonredribbon.org](http://pinkribbonredribbon.org/) | Internal search engine | "target product profile" | 0 | Title | 0 |  | **0** |  |
| 22/10/2018 | Unitaid | [https://unitaid.org](https://unitaid.org/) | Internal search engine | "target product profile" | 2 | Title | 1 | I only found newsletters and a potentially relevant article | **1** |  |
| 22/10/2018 | Malaria Consortium | <https://www.malariaconsortium.org/> | Internal search engine | "target product profile" | 15 | Title | 4 | Powerpoint presentations and newsletters were excluded | **4** |  |
| 22/10/2018 | ReAct Group | [https://www.reactgroup.org](https://www.reactgroup.org/) | Internal search engine | "target product profile" | 2 | Title | 0 | Records in the "News & Views" section were excluded | **0** |  |
| 22/10/2018 | DiaDev | [http://www.diadev.eu](http://www.diadev.eu/) | Internal search engine: "on the web" section | "target product profile" | 0 | Title | 0 | There is no internal search engine | **0** |  |
| 22/10/2018 |  | [http://www.diadev.eu](http://www.diadev.eu/) | Internal search engine: "presentation" section | "target product profile" | 0 | Title | 0 |  |  |  |
| 22/10/2018 |  | [http://www.diadev.eu](http://www.diadev.eu/) | Internal search engine: "academic publications" section | "target product profile" | 0 | Title | 0 |  | **0** |  |
| 22/10/2018 |  | [http://www.diadev.eu](http://www.diadev.eu/) | Internal search engine "working papers" section | "target product profile" | N/A | Title | N/A | I could not access this section. Blank page |  |  |
| 22/10/2018 |  | [http://www.diadev.eu](http://www.diadev.eu/) | Hand-searched in the "connections" section | "target product profile" | 0 | Title | 0 |  |  |  |
| 22/10/2018 |  | [http://www.diadev.eu](http://www.diadev.eu/) | Hand-searched "methods" section | "target product profile" | 2 | Title | 0 |  |  |  |
| 22/10/2018 |  | [http://www.diadev.eu](http://www.diadev.eu/) | Hand-searched "device case studies" section | "target product profile" | 0 | Title | 0 |  |  |  |
| 22/10/2018 | Public Health Emergency | <https://www.phe.gov/> | Internal search engine | "target product profile" | N/A | Title | N/A | The internal search engine is not functioning | **N/A** |  |
| 22/10/2018 | Galvmed | [https://www.galvmed.org](https://www.galvmed.org/) | Internal search engine | "target product profile" | 3 | Title | 0 | Reason for not including the records: TPP for animals | **0** |  |
| 22/10/2018 |  | <https://www.galvmed.org/resources/databases/document-repository/> | Internal search into Document Repository database | "target product profile" | 0 | Title | 0 |  | **0** |  |
| 22/10/2018 |  | <https://www.galvmed.org/resources/databases/livestock-laws-and-policy-database/> | Internal search into Livestock Livestock and Policy Database | "target product profile" | 119 | Title | 0 |  |  |  |
| 22/10/2018 | STP TB Partnership | [http://www.stoptb.org](http://www.stoptb.org/) | Internal search engine | "target product profile" | 48 | Title | 0 | Powerpoint presentations and newsletters were excluded *Google Custom Search | **1** | The number of included references entails relevant hits that were retrieved from both searches within the same website. Duplicates across both searches were deleted. |
| 22/10/2018 |  | [http://www.stoptb.org](http://www.stoptb.org/) | Hand-searched in "Technical Publications" section | "target product profile" |  | Title | 1 |  |  |  |
| 22/10/2018 | EMA | [https://www.ema.europa.eu](https://www.ema.europa.eu/) | Internal search engine | "target product profile" | 210 | Title | 0 | Search only for documents | **0** |  |
| 22/10/2018 | NICE | [https://www.nice.org.uk](https://www.nice.org.uk/) | Internal search engine | "target product profile" | 0 | Title | 0 |  | **0** |  |
| 22/10/2018 |  | [https://www.nice.org.uk](https://www.nice.org.uk/) | Internal search into the "Evidence search" engine | "target product profile" | 9 | Title | 0 |  | **0** |  |
| 22/10/2018 | IVCC | [http://www.ivcc.com](http://www.ivcc.com/) | Internal search engine | "target product profile" | 4 | Title | 0 | Only newsletters found | **0** |  |
| 22/10/2018 | Critical Path Institute | [https://c-path.org](https://c-path.org/) | Internal search engine | "target product profile" | 3 | Title | 1 |  | **1** |  |
| 22/10/2018 | European Directorate for the Quality of Medicines & HealthCare | <https://www.edqm.eu/> | Internal search engine | "target product profile" | 5 | Title | 0 |  | **0** |  |
| 22/10/2018 | AIGHD | <https://www.aighd.org/> | Search into the "Scientific Publications & PhD theses" | "target product profile" | N/A | Title | N/A | Blank page as a result of the search | **0** |  |
| 22/10/2018 |  | <https://www.aighd.org/> | Internal search engine | "target product profile" | 0 | Title | N/A |  |  |  |
| 22/10/2018 | IMPT for Reproductive Health | <https://www.theimpt.org/> | Search into "Resource Database" | "target product profile" | 3 | Title | 3 |  | **4** | The number of included references entails relevant hits that were retrieved from both searches within the same website. Duplicates across both searches were deleted. |
| 22/10/2018 |  | <https://www.theimpt.org/> | Internal search engine | "target product profile" | 11 | Title | 4 |  |  |  |
| 22/10/2018 | ICH harmonisation for better health | [https://www.ich.org](https://www.ich.org/) | Internal search engine | "target product profile" | 0 | Title | 0 |  | **0** |  |
| 22/10/2018 | Product Quality Research Institute | <http://pqri.org/> | Internal search engine | "target product profile" | 0 | Title | 0 |  | **0** |  |
| 22/10/2018 |  | <http://pqri.org/> | Search into "White papers" section | "target product profile" | 0 | Title | 0 |  |  |  |
