# Supplementary material for: Target Product Profiles for medical tests: a systematic review of current methods
Source: BMC Med. 2020 May 11;18:119. doi: 10.1186/s12916-020-01582-1 (PMC7212678; doi:10.1186/s12916-020-01582-1)
Supplement: Supplementary file 2 — Additional file 2. Test characteristics. [file 12916_2020_1582_MOESM2_ESM.docx]

# Additional File 2: Test characteristics

Table 2.1 Number and % of test characteristics reported by all TPPs (n=44), sorted by clusters [part 1]

| **Unmet clinical need** | | **Clinical validity** | |
| --- | --- | --- | --- |
|  | *n (%)* |  | n (%) |
| Intended use | 43 (98) | Diagnostic/testing sensitivity | 31 (70) |
| Medical need | 9 (20) | Diagnostic/testing specificity | 28 (64) |
| Target population | 33 (75) | Positive predictive value | 1 (2) |
| Target user | 33 (75) | Negative predictive value | 1 (2) |
| Medical decision to be influenced | 4 (9) | Field performance | 1 (2) |
| Fit with clinical workflow | 7 (16) | Precision/concordance | 5 (11) |
| Target level of health system | 38 (86) | False recent ratio (%) | 1 (2) |
| Test rationale | 2 (5) | (Generic) sensitivity | 12 (27) |
| Description of test concept | 2 (5) | (Generic) specificity | 12 (27) |
| Proof of concept | 1 (2) | Test performance with disease subgroups | 1 (2) |
|  |  | What is the risk of an inaccurate test results? | 1 (2) |
| **Analytical performance** | | | |
| Analytical performance |  | Platform throughput | 2 (5) |
| Analytical specificity | 18 (41) | Specimen/sample capacity and throughput | 17 (39) |
| Analytical sensitivity | 11 (25) | Manual sample/specimen preparation | 34 (77) |
| Strain specificity | 2 (5) | Overall sample preparation | 5 (11) |
| Limit of quantification/detection | 13 (30) | Need for operator to transfer a precise volume of sample | 7 (16) |
| Assay throughput | 9 (20) | Reagent integration/preparation | 8 (18) |
| Volume sample/specimen | 16 (36) | Reagent kit (transport, storage and stability, supplies not included in kit) | 3 (7) |
| Daily throughput (per module) | 3 (7) | Reagent kit reconstitution/packaging | 8 (18) |
| Assay design/format | 19 (43) | Sample type | 42 (95) |
| Reproducibility | 16 (36) | Control/comparative reference method | 17 (39) |
| Reproducibility near clinical threshold | 1 (2) | Target molecule to be detected | 17 (39) |
| Robustness | 1 (2) | Type of analysis | 8 (18) |
| Interferences | 5 (11) | Calibration | 19 (43) |
| Duration of valid sample | 5 (11) | Precision/concordance | 6 (14) |
| Duration of valid result | 7 (16) | Quality control | 15 (34) |
| Result stability | 1 (2) | Internal quality control | 16 (36) |
| In use stability | 7 (16) | External quality control | 4 (9) |
| Quantification/quantitation | 3 (7) | Indeterminate test results | 1 (2) |
| Multiplexing | 7 (16) | Time to test result | 38 (86) |
| Cross reactivity | 1 (2) | Device failure/invalid rate | 1 (2) |
| Kit quality indicators | 1 (2) | Test performance with disease groups | 1 (2) |
| (Generic) sensitivity | 12 (27) | (Generic) specificity | 12 (27) |
| Result | 4 (9) |  |  |
| **Costs** | | **Environmental impact** | |
| Price/cost of individual test | 27 (61) | Environmental footprint | 3 (7) |
| Cost per diagnosis | 2 (5) |  |  |
| (Capital) cost per instrument | 15 (34) |  |  |
| Table 2.1 Number and % of test characteristics reported by all TPPs (n=44), sorted by clusters [part 2] | | | |
| **Costs** | | **Clinical utility** | |
|  | n (%) |  | n (%) |
| Cost of platform to end user | 3 (7) | Intended outcome and linkage to care | 1 (2) |
| Cost of consumables | 3 (7) |  |  |
| Cost of manufacturing single use device | 3 (7) |  |  |
| Expected scale of manufacture | 4 (9) | What is the risk of an inaccurate test result? | 1 (2) |
| Potential market | 1 (2) | **Regulatory requirements** | |
| Market segmentation/channels to the market | 10 (23) | Regulatory requirements | 15 (34) |
| Region(s) of commercialisation | 11 (25) | Product registration path | 11 (25) |
| Competitive landscape | 1 (2) |  |  |
| **Human factors** | | | |
| Assay packaging | 4 (9) | Data analysis | 4 (9) |
| Data capture | 8 (18) | Data export ( connectivity and interoperability, electronics and software) | 18 (41) |
| Data handling | 2 (5) | Data input | 2 (5) |
| Hands-on time | 2 (5) | Instruction for use | 3 (7) |
| Labelling | 3 (7) | Language | 3 (7) |
| Materials used | 3 (7) | Patient identification capability | 5 (11) |
| Rate of errors in device interpretation | 1 (2) | Readout/reading system | 6 (14) |
| Result | 2 (5) | Result documentation-data display | 17 (39) |
| Safety precautions (biosafety requirements) | 12 (27) | Service and support | 10 (23) |
| Supplies needed | 5 (11) | Test outcome (nature) | 21 (48) |
| Tool format and complexity | 12 (27) | Training and education | 33 (75) |
| Unit size | 2 (5) | User interface | 4 (9) |
| User-induced failure rate | 1 (2) | Walkway operation | 2 (5) |
| Equipment-specific human factors | 7 (16) | Ease of test result interpretation | 10 (23) |
| Service and support | 10 (23) | Test size and weight | 4 (9) |
| Test size and portability | 20 (45) |  |  |
| **Infrastructural requirements** | | | |
| Ancillary supplies | 7 (16) | Assay packaging | 4 (9) |
| Biosafety requirements | 20 (45) | Clean water | 10 (23) |
| Cold chain | 11 (25) | Environmental tolerance of packaged test kit | 2 (5) |
| External maintenance | 1 (2) | Infrastructural requirements | 6 (14) |
| Instrument-infrastructural requirement | 12 (27) | Maintenance | 15 (34) |
| Additional third-party consumable | 5 (11) | Materials used | 2 (5) |
| Multiuse platform | 2 (5) | Need for additional equipment/test/spare parts | 10 (23) |
| Power requirements | 23 (52) | Reagent kit (transport, storage and stability, supplies not included in kit) | 8 (18) |
| Service and support | 11 (25) | Shipping conditions | 5 (11) |
| Storage conditions and shelf life | 31 (70) | Storage conditions prior utilisation | 1 (2) |
| Thermal tolerance of assay | 3 (7) | Waste disposal | 22 (50) |
| Supplies needed | 5 (11) | Operating conditions | 9 (20) |
| Temperature and humidity | 27 (61) | Stability during transport | 23 (52) |
