# Supplementary material for: Target Product Profiles for medical tests: a systematic review of current methods
Source: BMC Med. 2020 May 11;18:119. doi: 10.1186/s12916-020-01582-1 (PMC7212678; doi:10.1186/s12916-020-01582-1)
Supplement: Supplementary file 3 — Additional file 3. Results. [file 12916_2020_1582_MOESM3_ESM.docx]

# **Additional File 3: Results**

**Calculation inter-reviewer agreement rate (κ statistic)**

Title/Abstract screening

*Table 3.1 Title/Abstract screening inter-reviewer agreement rate calculation*

|  |  | BS | | |
| --- | --- | --- | --- | --- |
|  |  | Included | Excluded | Tot |
| PC | Included | 13 | 1 | 14 |
|  | Excluded | 0 | 275 | 275 |
|  | Tot | 13 | 276 | 291 |

$$p_{e}=\left[ \left( \frac{n_{1}}{n} \right)* \left( \frac{m_{1}}{n} \right) \right]+\left[ \left( \frac{n_{0}}{n} \right)*\left( \frac{m_{0}}{n} \right) \right]=\left[ \left( \frac{13}{289} \right)* \left( \frac{14}{289} \right) \right]+\left[ \left( \frac{276}{289} \right)*\left( \frac{275}{289} \right) \right]=91\%$$

$$p_{0}=\frac{\left( a+d \right)}{n}=\frac{\left( 13+275 \right)}{289}=100\%$$

$$\kappa=\frac{\left( p_{0}-p_{e} \right)}{\left( 1-p_{e} \right)}=\frac{\left( 100\%-91\% \right)}{\left( 1-91\% \right)}=96\%$$

Full text screening

*Table 3.2 Full text screening inter-reviewer agreement rate calculation*

|  |  | BS | | |
| --- | --- | --- | --- | --- |
|  |  | Included | Excluded | Tot |
| PC | Included | 44 | 0 | 44 |
|  | Excluded | 1 | 63 | 64 |
|  | Tot | 45 | 63 | 108 |

$$p_{e}=\left[ \left( \frac{n_{1}}{n} \right)* \left( \frac{m_{1}}{n} \right) \right]+\left[ \left( \frac{n_{0}}{n} \right)*\left( \frac{m_{0}}{n} \right) \right]=\left[ \left( \frac{45}{108} \right)* \left( \frac{44}{108} \right) \right]+\left[ \left( \frac{63}{108} \right)*\left( \frac{64}{108} \right) \right]=91\%$$

$$p_{0}=\frac{\left( a+d \right)}{n}=\frac{\left( 44+63 \right)}{108}=99\%$$

$$\kappa=\frac{\left( p_{0}-p_{e} \right)}{\left( 1-p_{e} \right)}=\frac{\left( 100\%-99\% \right)}{\left( 1-99\% \right)}=96\%$$

*Table 3.3 Input sources reported to be used in scoping and drafting phase and stakeholders contributing to each phase*

|  | **Scoping** | **Drafting** |  |
| --- | --- | --- | --- |
|  | **n (%)^a^** | **n (%)^b^** |  |
| **TPPs reporting information on input sources considered** | 22 (50) | 33 (75) |  |
| **Input sources reported to be used in each phase** |  |  |  |
| Literature | 8 (36) | 22 (67) |  |
| Expert opinion | 15 (68) | 24 (73) |  |
| Meeting input | 4 (18) | 8 (24) |  |
| Consensus-meeting input | 0 (0) | 3 (9) |  |
| Available data | 1 (5) | 7 (21) |  |
| Interviews/Questionnaire with experts | 4 (14) | 2 (6) |  |
| Laboratory evaluations | 0 (0) | 1 (3) |  |
| Models | 0 (0) | 9 (27) |  |
| Guidelines | 0 (0) | 6 (18) |  |
| Early usability studies | 0 (0) | 1 (1) |  |
| Industry standard | 0 (0) | 1 (3) |  |
| Field observations | 1 (5) | 5 (15) |  |
| Ethnographic interviews | 1 (5) | 0 (0) |  |
| Reports | 2 (9) | 0 (0) |  |
| Policies | 1 (5) | 0 (0) |  |
| Market analyses | 1 (5) | 0 (0) |  |
| Websites of developers | 1 (5) | 0 (0) |  |

The percentages in relation to input sources do not add up to 100% because each TPP could choose more than one source.

^a^ Percentages are calculated in relation to the number of TPPs which provided information on input sources used during the scoping phase (n=22).

^b^ Percentages are calculated in relation to the number of TPPs which provided information on input sources used during the drafting phase (n=33).

Table 3.4 Extended summary table data extraction: publication format, disease area, funding body, test, aim TPP, decision-making phases described, description of decision-making process, input sources) to inform TPP and stakeholder involved for each included TPP (n=44)

| Authors | Publication format | Disease area | Funding body | Test(s) | Aim | Decision-making phases described | Decision-making process | Input source(s) to inform TPP^^[[1]](#footnote-1)^^ | Stakeholder(s) involved |
| --- | --- | --- | --- | --- | --- | --- | --- | --- | --- |
| **Chua *et al***. 2017 *(21)* | Journal article | Zika virus | /^[[2]](#footnote-2)^ | Diagnostic test for Zika infection and blood bank testing | / | -Drafting,  -Consensus-building | -Meeting with experts,  -Draft TPP,  -Consensus meeting,  -Revision following feedback | Meeting input (D),  Literature (D) | Researchers (D),  International public organisation(D)  Scientific associations (D) |
| **Denkinger *et al***. 2015 *(22)* | Journal article | Tubercolosis | Bill and Melinda Gates Foundation; American Society of Tropical Medicine and Hygiene; National Institute of Allergy and Infectious Diseases, National Institutes of Health, Department of Health and Human Services; | A molecular drug-susceptibility test | -To define minimal test characteristic as lowest acceptable specification  - To define optimal test characteristic as ideal value for that characteristic | -Scoping,  -Drafting  -Consensus-building | -Mapping,  -Survey to measure stakeholders’ preferences,  -Landscaping exercise,  -Draft TPP,  -Round of revisions,  -Shortened TPP draft,  -Presentation of shortened TPP to stakeholders,  -Consensus meeting | Literature (S,D, diagnostic accuracy^[[3]](#footnote-3)^)  Expert opinion (S,D),  Models (D, diagnostic accuracy),  Data available (S,D),  Market analyses (S) | Researchers (S),  Clinicians (S,D,C),  Policy makers (C),  Industry representatives (S),  Laboratory experts (S,D),  Implementers (C),  Representatives of national disease programs (C) |
| **Denkinger *et al***. 2015 *(23)* | Journal article | Tubercolosis | Bill and Melinda Gates Foundation | 1) A diagnostic test  2) A diagnostic biomarker test  3) A referral screening test | / | -Scoping  -Drafting  -Consensus-building | -Definition problem statement, -Priority-setting,  - Draft TPP,  -Round of revisions,  -Shortened draft TPP,  -Presentation of shortened TPP to stakeholders,  -Delphi-like survey to gauge stakeholders’ agreement with the TPP,  -Consensus meeting | Literature (S,D, diagnostic accuracy),  Expert opinion (S,D)  Reports (S)  Models (D, diagnostic accuracy) | Representatives of national disease programs (S,C),  Clinicians (S,D,C),  Researchers (S,D),  Laboratory experts (S),  Patient advocates (S),  Modelers (S),  Market experts (S),  Policy makers (D),  Industry representatives (D,C),  Technical/funding agencies (D) |
| **DIAMETER Project and PATH** 2014 *(24)* | Report | Malaria | Bill and Melinda Gates Foundation | A POC infection detection test | / | / | / | Literature (D, analytical accuracy^[[4]](#footnote-4)^),  Available data (D),  Field observation (D),  Models (D),  Expert opinion (analytical accuracy) | / |
| **Ding. *et al***. 2017 *(25)* | Journal article | Malaria | Department of Foreign Affairs and Trade, Australia | 1) A diagnostic test  2) A POC diagnostic test 3) A screening test | -To define minimal test characteristic as a value set to provide a distinguishing advantage  - To define minimal test characteristic as a value that provides optimal diagnostic effectiveness | -Scoping,  -Drafting  -Consensus-building | -Definition problem statement,  -Draft TPP,  -Round of revisions TPP,  -Survey to measure stakeholders’ preferences | Literature (D, analytical accuracy),  Meeting input (S),  Expert opinion (S,D)  Models (D,analytical accuracy) | Researchers (S,C),  Representatives of national disease programs (S,C),  International public organisation (S,C) |
| **Dittrich,. *et a****l*. 2016 *(26)* | Journal article | Malaria | The Dutch Government, with aid from the UK and the Australian Government | A test to distinguish bacterial from non-bacterial infections | -To define minimal test characteristic as lowest acceptable specification  - To define optimal test characteristic as ideal value for that characteristic | -Scoping,  -Drafting,  -Consensus-building | -Landscaping exercise,  -Draft TPP,  -Delphi-like approach to gauge stakeholders’ agreement with the TPP,  -Round of revisions,  -Revision following  feedback ,  -Priority-setting,  -Consensus meeting | Literature (S,D),  Expert opinion (D, analytical accuracy),  Industry standard (D) | Researchers (D,C),  Laboratory experts (D,C) ,  Microbiologists (D,C),  Health economists (D,C),  Industry representatives (D,C),  International public organisation (D,C) |
| **Donadeu, *et al***. 2017 *(27)* | Journal article | Taenia solium taeniasis, neurocysticercosis and porcine cysticercosis | / | 1) A POC test that could be used for surveillance  2) A specific test  3)A POC test 4) A monitoring test | / | -Scoping,  -Drafting,  -Consensus-building | -Introductory meeting  -Identification of most important needs,  -Draft TPP,  -Survey to measure stakeholders’ preferences,  -Revision following feedback,  -Round of revisions | Literature (D, analytical accuracy)  Expert opinion (D, analytical accuracy),  Meeting input (S) | International public organisation (S,C),  Representatives of national disease programs (C),  Policy-makers (C)  Non-profit sector (C)  Experts (unspecific) (C),  Industry representatives (C),  Researchers (C),  Clinicians (C) |
| **Ebels *et*** *al*. 2014 *(10)* | Journal article | Malaria | / | A combined test | No minimal/optimal test characteristic reported | -Scoping,  -Drafting | -Definition problem statement,  -Consolidation of findings,  -Draft TPP | Interviews with experts (D),  Field observations (S,D),  Ethnographic interviews (S) | Industry representatives (S,D),  Researchers (S,D),  Clinicians (S,D)  Strategists (S,D),  Laboratory experts (S,D),  Microbiologists (S,D),  Program manager (S,D),  International public organisation (S,D),  Donors (S,D),  Representatives of national disease programs (S,D),  Policy makers (S,D) |
| **FIND** 2017 *(29)* | Report | HIV Infection | Bill and Melinda Gates Foundation | A diagnostic test | / | -Scoping,  -Drafting  -Consensus-building | -Definition problem statement,  -Identification of most important needs,  -Draft TPP,  -Consolidation of findings,  -Draft TPP shortened,  -Revision following feedback,  -Delphi-like approach to gauge stakeholders’ agreement with the TPP,  -Consensus meeting | Expert opinion (S,D),  Meeting input (D) | -Researcher (S,D,C),  -Industry representatives (S,C),  -International public organisations (S,D,C),  -Policy-makers (S,D,C),  -Scientific associations (S,D,C) |
| **FIND** 2017 *(30)* | Report | Human African trypanosomiasis | / | Rapid test for diagnosis and screening | -To define minimal test characteristic as lowest acceptable specification  - To define optimal test characteristic as ideal value for that characteristic | -Drafting,  -Consensus-building | -Draft TPP,  -Delphi-like approach to gauge stakeholders’ agreement with the TPP | / | Researchers (C),  Non-profit sector (C),  Industry representatives (C) |
| **FIND** 2017 *(31)* | Report | Human African trypanosomiasis | / | A screening test | -To define minimal test characteristic as lowest acceptable specification  - To define optimal test characteristic as ideal value for that characteristic | -Drafting,  -Consensus-building | -Draft TPP,  -Delphi-like approach to gauge stakeholders’ agreement with the TPP | / | Researchers (C),  International public organisation (C),  Non-profit sector (C),  Industry representatives (C) |
| **FIND** **and Forum for Collaborative HIV Research** 2015 (28) | Report | Hepatits C | / | 1) HCV nucleic acid amplification diagnostic test 2) HCV cAg diagnostic test | -To define minimal test characteristic as lowest acceptable specification  - To define optimal test characteristic as ideal value for that characteristic | -Scoping,  -Drafting,  -Consensus-building | -Priority-setting,  -Draft TPP,  -Draft TPP shortened,  -Delphi-like approach to gauge stakeholders’ agreement with the TPP,  -Consensus meeting | Literature (D, analytical accuracy),  Expert opinion (S,D, accuracy),  Available data (analytical accuracy)  Models (D, diagnostic accuracy) | Clinicians (C),  Implementers (C),  Representatives of national disease programs (C),  Industry representatives (C),  Technical/funding agencies (C),  Patient advocates (C),  International public organisation (C),  Researchers (C) ,  Other (C) |
| **Gal *et al***. 2018 *(32)* | Journal article | Community-acquired lower respiratory tract infection | Innovative Medicines Initiative IMI-JU-02-2009-04 Infectious Diseases-Diagnostic Tools | POC test | -To define minimal test characteristic as lowest acceptable specification  - To define optimal test characteristic as ideal value for that characteristic | -Scoping,  -Drafting  -Consensus-building | -Priority-setting,  -Survey to retrieve input for TPP ,  -Meeting with stakeholders,  -Draft TPP,  -Revision following feedback | Literature (D, diagnostic accuracy),  Expert opinion (S,D),  Meeting input (D), | Clinicians (S,D,C),  Microbiologists (S,C),  Industry representatives (S,D,C),  Researchers (S,D,C),  Market experts (S,D,C), |
| **International Diagnostics Centre (IDC)** 2013 *(33)* | Published TPP table | HIV | / | A POC test | / | / | / | / | / |
| **International Diagnostics Centre (IDC)** 2013 *(34)* | Published TPP table | HIV | / | A POC test | / | / | / | / | / |
| **International Diagnostics Centre (IDC)** 2013 *(35)* | Published TPP table | HIV | / | A POC test | / | / | / | / | / |
| **Internationa Diagnostics Centre (IDC)** 2014 *(36)* | Published TPP table | HIV and Syphilis | / | A combined test | / | / | / | / | / |
| **Lim *et al***. 2018 *(37)* | Journal article | Soil-transmitted helminths | / | 1) A diagnostic test for mapping/monitoring  2) A diagnostic test to confirm decision to stop intervention | - To define minimal test characteristics as “must have” requirements  - To define optimal characteristics as “nice to have” requirements | -Scoping,  -Drafting  -Consensus | -Landscaping exercise ,  -Definition problem statement,  -Draft TPP,  Round of revisions,  -Meeting with stakeholders,  -Revision following feedback | Literature (S,D, diagnostic accuracy)  Expert opinion (S,D),  Guidelines (D),  Meeting input (D) | Researchers (S,D,C),  Implementers (S,D,C),  Industry representatives (S,D,C) |
| **Nsanzabana *et al***. 2018 *(38)* | Journal article | Antimalarial drug resistance | / | Antimalarial drug resistance diagnostic test | -To define minimal test characteristic as lowest acceptable specification  - To define optimal test characteristic as ideal value for that characteristic | -Scoping,  -Drafting  -Consensus-building | -Landscape analysis,  -Draft TPP,  - Delphi-like approach to gauge stakeholders’ agreement with the TPP,  -Consensus-meeting,  - Revision following feedback | Literature (S,D, analytical accuracy), | Researchers (C),  International public organisation (C),  Industry representatives (C) |
| **Pal *et al***. 2016 *(39)* | Journal article | Dengue fever | Military Infectious Diseases Research Program, U.S. Army Medical Research and  Materiel Command | A diagnostic test | / | -Scoping,  -Drafting | -Identification of most important needs,  -Draft TPP | Expert opinion (D, diagnostic accuracy),  Models (D, diagnostic accuracy),  Questionnaire provided to expert (S) | Experts (unspecific) (S,D),  Other (S,D) |
| **PATH** 2014 *(9)* | Report | HIV | / | A self-test | -To define minimal test characteristic as lowest acceptable specification  - To define optimal test characteristic as ideal value for that characteristic | -Scoping,  -Drafting | -Draft TPP,  -Round of revisions | Literature (D, analytical accuracy),  Expert opinion (D),  Available data (D),  Early usability studies (D, analytical accuracy),  Laboratory evaluations (D, analytical accuracy) | / |
| **PATH** 2015 *(40)* | Report | Neglected Tropical Diseases | Bill and Melinda Gates Foundation;  UK Department for International Development | 1) Later flow tools  2) Nucleic acid amplification tools | -To define minimal test characteristic as lowest acceptable specification  - To define optimal test characteristic as ideal value for that characteristic | -Scoping,  -Drafting,  -Consensus-building | -Definition problem statement,  -Draft TPP,  -Revision following feedback,  -Round of revisions | Literature (S),  Interviews with experts (S)  Expert opinion (D) | Industry representatives (S,D),  Researchers (S,D),  Representatives of national disease programs (S,D),  Non-profit sector (S,D),  Policy makers (S,D),  Donors (S,D) |
| **PATH** 2015 *(41)* | Report | Schistosomiasis | / | A rapid diagnostic test | -To define minimal test characteristic as a must-have  -To define optimal test characteristic as an ideal value that would make a tool more valuable | / | / | Literature (D, analytical accuracy),  Guidelines (D),  Expert opinion (D),  Field observation (D),  Available data (D) | / |
| **PATH** 2015 *(42)* | Report | Schistosomiasis | / | A lateral flow test | -To define minimal test characteristic as a must-have  -To define optimal test characteristic as an ideal value that would make a tool more valuable | / | / | Literature (D, analytical accuracy),  Expert opinion (D),  Field observation (D),  Guidelines (D),  Available data (D) | / |
| **PATH** 2015 *(43)* | Report | Schistomiasis | / | A lateral flow test | -To define minimal test characteristic as a must-have  -To define optimal test characteristic as an ideal value that would make a tool more valuable | / | / | Literature (D, analytical accuracy)  Expert opinion (D),  Field observation (D),  Guidelines (D),  Available data (D) | / |
| **PATH** 2015 *(44)* | Report | Trachoma | / | A lateral flow rapid diagnostic test | -To define minimal test characteristic as a must-have  -To define optimal test characteristic as an ideal value that would make a tool more valuable | / | / | Literature (D, analytical accuracy),  Guidelines (D),  Available data (D),  Expert opinion (D) | / |
| **PATH** 2015 *(45)* | Report | Trachoma | / | A lateral flow rapid diagnostic test | -To define minimal test characteristic as a must-have  -To define optimal test characteristic as an ideal value that would make a tool more valuable | / | / | Literature (D, analytical accuracy),  Guidelines (D),  Available data (D),  Expert opinion (D) | / |
| **Peck *et al***. 2015 *(46)* | Report | Yellow fever | Global Alliance for Vaccines and Immunisation Fund | A field-deployable test | / | -Scoping,  -Drafting, | -Definition problem statement,  -Draft TPP | Interviews with experts (S,D) | Researchers (S,D),  Industry representatives (S,D)  Clinicians (S,D),  Technical/funding agencies (S,D),  Other (S,D) |
| **Porras et al**. 2015 *(47)* | Journal article | Chagas disease | Pan-American Health Organisation; The Special Programme for Research and Training in Tropical Diseases | 1-2) a POC diagnostic test 3) a treatment-monitoring test | / | -Scoping,  -Drafting, | -Meeting with stakeholders,  -Definition problem statement,  -Draft TPP | Meeting input (S,D)  Expert opinion (S) | / |
| **Reipold *et al***. 2017 *(48)* | Journal article | Hepatitis C | WHO | A diagnostic test | -To define minimal test characteristic as lowest acceptable specification  - To define optimal test characteristic as ideal value for that characteristic | -Scoping,  -Drafting,  -Consensus-building | -Identification of stakeholders to involve in draft TPP ,  -Priority-setting,  -Definition TPP domains ,  - Draft TPP,  -Delphi-like approach to gauge stakeholders’ agreement with the TPP,  -Consensus meeting  -Survey to measure stakeholders’ preferences, | Literature (D, analytical accuracy, diagnostic accuracy),  Expert opinion (S,D, diagnostic accuracy),  Available data (analytical accuracy)  Models (D, diagnostic accuracy) | International public organisation (S,D,C),  Researchers (S,D,C),  Technical/funding agencies (S,D,C),  Industry representatives (S,D,C),  Patient advocates (S,D,C),  Representatives of national disease programs (S,D,C),  Clinicians (S,D,C),  Implementers (S,D,C)  Policy-makers (C)  Program managers (C) |
| **Solomon *et al***. 2012 (49) | Journal article | Neglected tropical diseases | WHO | 1) A diagnostic tool with mapping and impact monitoring potential  2) A diagnostic tool | / | -Drafting | -Draft TPP,  - Consensus-meeting | Consensus meeting inputs (D) | Laboratory experts (D,C),  Modelers (D,C),  Health economists (D,C),  Experts (unspecific) (D,C),  Program manager (D,C) |
| **Toskin *et al***. 2017 (50) | Journal article | Sexually transmitted infections | / | 1) A combined diagnostic POC  2) POC platforms  3) A combined diagnostic test | / | -Scoping,  -Drafting,  -Consensus-building | -Landscaping exercise ,  -Reviewing available literature and data,  -Survey to retrieve input for TPP ,  -Draft TPP,  -Consensus meeting,  -Revision following feedback,  -Round of revisions | Literature (S,D),  Models (D) ,  Expert opinion (D), Consensus meeting inputs (D, accuracy),  Reports (S),  Policies (S),  Websites of developers (S),  Interviews with experts (S) | Clinicians (D,C),  Industry representatives (S,D),  International public organisation (D,C),  Experts (unspecific) (D,C),  Laboratory experts (D,C),  Microbiologists (D,C) |
| **UNICEF** 2014 (51) | Report | Pneumonia Acute Respiratory infection | / | A diagnostic aid | / | -Scoping,  -Drafting,  -Consensus-building | -Defining scope TPP ,  -Draft TPP,  -Round of revisions,  -Survey to measure stakeholders’ preferences | Literature (S,D)  Expert opinion (D) | Researchers (S,D,C),  Industry representatives (S,D,C),  International public organisation (S,D,C), |
| **UNICEF** 2016 *(52)* | Report | E.coli infection | / | Water quality testing product | / | / | / | / | / |
| **UNICEF** 2017 *(53)* | Report | E.coli infection | / | Water quality testing product | / | / | -Survey to measure stakeholders’ preferences | / | / |
| **UNICEF** 2017 *(54)* | Report | Zika virus | / | A diagnostic test | / | -Scoping,  -Drafting | -Defining scope TPP,  -Draft TPP,  -Round of revisions,  -Revision following feedback | Literature (D),  Expert opinion (S,D) | Researchers (S,D),  Industry representatives (S,D),  International public organisation (S,D), |
| **Utzinger *et al***. 2015 *(55)* | Journal article | Schistosomiasis | Bill and Melinda Gates Foundation,  European Union’s Seventh Framework Programme for research, technological development and demonstration | 1) A diagnostic test for mapping-monitoring  2) A diagnostic tool | No minimal test characteristic reported | / | / | / | / |
| **WHO** 2014 *(60)* | Report | Tubercolosis | Bill and Melinda Gates Foundation | 1) A diagnostic test  2) A diagnostic biomarker test 3) A referral screening test 4) A drug-susceptibility test | -To define minimal test characteristic as lowest acceptable specification  - To define optimal test characteristic as ideal value for that characteristic | -Scoping,  -Drafting,  -Consensus-building | -Priority-setting,  -Draft TPP,  -Draft TPP shortened,  -Delphi-like approach to gauge stakeholders’ agreement with the TPP,  -Consensus meeting | Literature (D, analytical accuracy, diagnostic accuracy),    Expert opinion (S,D, analytical accuracy, diagnostic accuracy),  Consensus meeting inputs (D),  Models (D, analytical accuracy, diagnostic accuracy) |  |
| **WHO** 2016 *(61)* | Report | Meningits | / | A rapid diagnostic test | / | / | / | / |  |
| **WHO and FIND** 2017 *(58)* | Report | Tubercolosis | / | A test for predicting disease progression | / | -Draft,  -Consensus-building | -Meeting with stakeholders ,  -Draft TPP,  -Survey to retrieve input for TPP ,  -Delphi-like approach to gauge stakeholders’ agreement with the TPP,  -Consensus meeting | Expert opinion (D),  Meeting input (D) |  |
| **WHO** 2018 *(59)* | Report | Buruli ulcer | / | 1) A rapid diagnostic test 2) A diagnostic test with treatment-monitoring potential | / | -Scoping,  -Draft | -Definition problem statement,  -Draft TPP | Expert opinion (S)  Meeting input (D) |  |
| **WHO** 2016 *(56)* | Report | Taenia solium taeniasis/ cysticercosis | / | 1) A human copro Ag-taeniasis test,  2) A combined human Ag/Ab-cysticercosis test  3) A porcine Ag-cysticercosis test | / | -Scoping,  -Draft | -Definition problem statement,  -Draft TPP | Meeting inputs (D),  Expert opinion (S) |  |
| **WHO, FIND and Medecins sans Frontiers** 2017 *(8)* | Report | Severe febrile illness | WHO | A diagnostic platform | / | -Draft,  -Consensus-building | -Draft TPP,  -Delphi-like approach to gauge stakeholder’ agreement with the TPP,  -Revision following feedback,  -Consensus meeting | Expert opinion (D) |  |
| **WHO** 2014 *(57)* | Report | Ebola virus | / | A rapid test | / | / | / | / |  |

*Table 3.5 Transparency assessment. TPPs sorted from the most transparent to the least transparent*

|  | **Study mentions type of sources** | **Study quotes specific literature** | **Study mentions type of sources for scoping phase** | **Study describes decision-making steps** | **Study reports types of stakeholders** | **Study lists the name of the organizations stakeholders are part of** | **Study lists the name of each stakeholders involved** | **Study reports the rationale for inviting certain stakeholders** | **Study specifies funding body** | **Total score** |
| --- | --- | --- | --- | --- | --- | --- | --- | --- | --- | --- |
| Nsanzabana, et al. (38) | ✓ | ✓ | ✓ | ✓ | ✓ | ✓ | ✓ | ✓ | 🗶 | 8 |
| WHO. (60) | ✓ | ✓ | ✓ | ✓ | ✓ | ✓ | ✓ | 🗶 | ✓ | 8 |
| Dittrich, et al. (26) | ✓ | ✓ | ✓ | ✓ | ✓ | 🗶 | 🗶 | ✓ | ✓ | 7 |
| FIND and Forum for Collaborative HIV Research (28) | ✓ | ✓ | ✓ | ✓ | ✓ | ✓ | ✓ | 🗶 | 🗶 | 7 |
| Lim, et al. (37) | ✓ | ✓ | ✓ | ✓ | ✓ | ✓ | ✓ | 🗶 | 🗶 | 7 |
| Denkinger, et al. (22) | ✓ | ✓ | ✓ | ✓ | ✓ | 🗶 | 🗶 | 🗶 | ✓ | 6 |
| Reipold, et al. (48) | ✓ | ✓ | ✓ | ✓ | ✓ | 🗶 | 🗶 | 🗶 | ✓ | 6 |
| Toskin, et al. (50) | ✓ | 🗶 | ✓ | ✓ | ✓ | ✓ | ✓ | 🗶 | 🗶 | 6 |
| FIND. (29) | ✓ | 🗶 | ✓ | ✓ | 🗶 | ✓ | ✓ | 🗶 | ✓ | 6 |
| WHO and FIND. (58) | ✓ | 🗶 | 🗶 | ✓ | ✓ | ✓ | ✓ | ✓ | 🗶 | 6 |
| WHO and TDR. (56) | ✓ | 🗶 | ✓ | ✓ | ✓ | ✓ | ✓ | 🗶 | 🗶 | 6 |
| Peck, et al. (46) | ✓ | 🗶 | ✓ | ✓ | ✓ | 🗶 | 🗶 | ✓ | ✓ | 6 |
| Denkinger, et al. (23) | ✓ | 🗶 | ✓ | ✓ | ✓ | 🗶 | 🗶 | 🗶 | ✓ | 5 |
| Ding, et al. (25) | ✓ | 🗶 | ✓ | ✓ | ✓ | 🗶 | 🗶 | 🗶 | ✓ | 5 |
| Gal, et al. (32) | ✓ | 🗶 | ✓ | ✓ | ✓ | 🗶 | 🗶 | 🗶 | ✓ | 5 |
| PATH. (40) | ✓ | 🗶 | ✓ | ✓ | ✓ | 🗶 | 🗶 | 🗶 | ✓ | 5 |
| WHO and FIND. (59) | ✓ | 🗶 | ✓ | ✓ | 🗶 | ✓ | ✓ | 🗶 | 🗶 | 5 |
| Pal, et al. (39) | ✓ | 🗶 | ✓ | ✓ | ✓ | 🗶 | 🗶 | 🗶 | ✓ | 5 |
| Porras, et al. (47) | ✓ | 🗶 | 🗶 | ✓ | 🗶 | ✓ | 🗶 | 🗶 | ✓ | 4 |
| Donadeu, et al. (27) | ✓ | 🗶 | ✓ | ✓ | ✓ | 🗶 | 🗶 | 🗶 | 🗶 | 4 |
| PATH. (9) | ✓ | ✓ | 🗶 | ✓ | 🗶 | 🗶 | 🗶 | 🗶 | ✓ | 4 |
| UNICEF. (54) | ✓ | 🗶 | ✓ | ✓ | ✓ | 🗶 | 🗶 | 🗶 | 🗶 | 4 |
| UNICEF. (51) | ✓ | 🗶 | ✓ | ✓ | ✓ | 🗶 | 🗶 | 🗶 | 🗶 | 4 |
| Ebels, et al. (10) | ✓ | 🗶 | ✓ | ✓ | ✓ | 🗶 | 🗶 | 🗶 | 🗶 | 4 |
| Chua, et al. (21) | ✓ | 🗶 | 🗶 | ✓ | ✓ | 🗶 | 🗶 | 🗶 | 🗶 | 3 |
| DIAMETER Project and PATH. (24) | ✓ | ✓ | 🗶 | 🗶 | 🗶 | 🗶 | 🗶 | 🗶 | ✓ | 3 |
| Solomon, et al. (49) | ✓ | 🗶 | 🗶 | 🗶 | ✓ | 🗶 | 🗶 | 🗶 | ✓ | 3 |
| FIND. (30) | 🗶 | 🗶 | 🗶 | ✓ | ✓ | 🗶 | 🗶 | 🗶 | 🗶 | 2 |
| PATH. (41) | ✓ | ✓ | 🗶 | 🗶 | 🗶 | 🗶 | 🗶 | 🗶 | 🗶 | 2 |
| PATH. (43) | ✓ | ✓ | 🗶 | 🗶 | 🗶 | 🗶 | 🗶 | 🗶 | 🗶 | 2 |
| PATH. (45) | ✓ | ✓ | 🗶 | 🗶 | 🗶 | 🗶 | 🗶 | 🗶 | 🗶 | 2 |
| FIND. (31) | 🗶 | 🗶 | 🗶 | ✓ | ✓ | 🗶 | 🗶 | 🗶 | 🗶 | 2 |
| PATH. (44) | ✓ | ✓ | 🗶 | 🗶 | 🗶 | 🗶 | 🗶 | 🗶 | 🗶 | 2 |
| PATH. (42) | ✓ | ✓ | 🗶 | 🗶 | 🗶 | 🗶 | 🗶 | 🗶 | 🗶 | 2 |
| WHO, et al. (8) | ✓ | 🗶 | 🗶 | ✓ | 🗶 | 🗶 | 🗶 | 🗶 | 🗶 | 2 |
| WHO and FIND. (57) | 🗶 | 🗶 | 🗶 | 🗶 | 🗶 | ✓ | 🗶 | 🗶 | 🗶 | 1 |
| Utzinger, et al. (55) | 🗶 | 🗶 | 🗶 | 🗶 | 🗶 | 🗶 | 🗶 | 🗶 | ✓ | 1 |
| UNICEF. (52) | 🗶 | 🗶 | 🗶 | 🗶 | 🗶 | 🗶 | 🗶 | 🗶 | 🗶 | 0 |
| UNICEF. (53) | 🗶 | 🗶 | 🗶 | 🗶 | 🗶 | 🗶 | 🗶 | 🗶 | 🗶 | 0 |
| WHO. (61) | 🗶 | 🗶 | 🗶 | 🗶 | 🗶 | 🗶 | 🗶 | 🗶 | 🗶 | 0 |
| International Diagnostics Centre (IDC) (26) | 🗶 | 🗶 | 🗶 | 🗶 | 🗶 | 🗶 | 🗶 | 🗶 | 🗶 | 0 |
| International Diagnostics Centre (IDC) (27) | 🗶 | 🗶 | 🗶 | 🗶 | 🗶 | 🗶 | 🗶 | 🗶 | 🗶 | 0 |
| International Diagnostics Centre (IDC) (28) | 🗶 | 🗶 | 🗶 | 🗶 | 🗶 | 🗶 | 🗶 | 🗶 | 🗶 | 0 |
| International Diagnostics Centre (IDC) (29) | 🗶 | 🗶 | 🗶 | 🗶 | 🗶 | 🗶 | 🗶 | 🗶 | 🗶 | 0 |

1. *S= scoping phase; D= Drafting phase ; C= Consensus-building phase*  [↑](#footnote-ref-1)
2. */ = No information* [↑](#footnote-ref-2)
3. *Input source used to inform diagnostic accuracy* [↑](#footnote-ref-3)
4. *Input source used to inform analytical accuracy* [↑](#footnote-ref-4)
